# Saussurea costus alleviates ulcerative colitis by regulating the gut microbiota and improving intestinal barrier integrity

**DOI:** 10.3389/fcimb.2025.1528578

**Published:** 2025-01-28

**Authors:** Wen-lin Pang, Tian-gang Li, Yin-ying Wang, Li-yun Song, Li Li, Xiao-ya Li, Yong Qiu, Zhong-shan Yang

**Affiliations:** ^1^ Yunnan Provincial Key Laboratory of Integrated Traditional Chinese and Western Medicine for Chronic Disease in Prevention and Treatment, Yunnan University of Traditional Chinese Medicine, Kunming, Yunnan, China; ^2^ Zhongshan Hospital of Traditional Chinese Medicine, Affiliated to Guangzhou University of Chinese Medicine, Zhongshan, Guangdong, China; ^3^ Yunnan Provincial Department of Education, Engineering Research Center of Classic Formula Regulate Immunity in Chronic Disease Prevention and Treatment, Kunming, Yunnan, China

**Keywords:** gut microbiota, intestinal barrier, inflammation, saussurea costus, ulcerative colitis

## Abstract

**Introduction:**

The global health challenge of ulcerative colitis (UC) has been classified by the WHO as a modern refractory disease, commonly referred to as green cancer, with limited treatment options still available, highlighting the urgent need for the development of new therapeutic strategies. Recent pharmacological research has shown that traditional Chinese medicine saussurea costus (SC) possesses beneficial antibacterial and anti-inflammatory properties. Nevertheless, its underlying mechanism remains elusive.

**Methods:**

Firstly, we identified the main active components of SC through UHPLC-QTOF-MS analysis. Subsequently, UC mice were induced using DSS and administered different doses of SC to evaluate its efficacy. Additionally, the impact of SC on the repair of the intestinal mucosal barrier was evaluated through immunofluorescence and western blot. Furthermore, 16s rRNA gene sequencing was conducted to elucidate the contribution of gut microbiota to UC pathogenesis.

**Results:**

The primary components of SC include Proline, Phenylalanine, Isoleucine, Lucidenic acid M, and Pyroglutamic acid. The efficacy of SC was concurrently assessed, revealing its potential to ameliorate histological injury in colitis mice. Furthermore, SC was found to decrease levels of TNF-α, IL-1β, IL-8, and IL-18 while promoting the expression of IL-10 and IL-22. Similarly, we also found that the expression of ZO-1 and Occludin was reversed by SC in colitis mice. In addition, analysis of 16S rRNA gene sequencing indicated that SC reduced harmful bacterial populations, such as *Proteobacteria*, while simultaneously enhancing the levels of beneficial bacteria like *Lactobacillus*, thereby contributing to the improvement of UC pathology.

**Conclusion:**

This study highlights the therapeutic potential of SC in managing UC through its ability to attenuate inflammatory responses, restore intestinal barrier functionality, and modulate gut microbiota composition, which findings offer insights into potential strategies for advancing UC treatment.

## Graphical Abstract

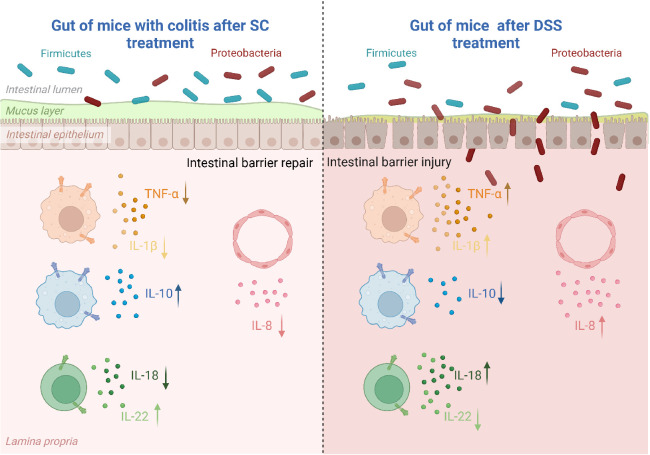

## 1 Introduction

Chronic inflammation of the colonic and rectal mucosa that recurs is the primary characteristic of ulcerative colitis (UC) (Xia et al., 2024). Over the past decade, UC has emerged as a public health challenge worldwide. In 2023, the prevalence of UC was estimated to be 5 million cases around the world (Le Berre et al., 2023), and the incidence continues to rise rapidly across South America, Eastern Europe, Asia, and Africa (Ng et al., 2017). Nevertheless, the pathogenesis of UC has not been fully elucidated, the development of UC is influenced by a variety of factors, encompassing genetic predisposition, gut microbiota dysbiosis, and environmental factors (Kobayashi et al., 2020). There is growing evidence that UC is an overly robust mucosal immune response to specific gut microbiota dysregulation, characterized by abnormal microbiota composition and bacterial products (Fang et al., 2021). The maintenance of intestinal mucosal homeostasis depends on the balanced coexistence of a diverse commensal microbial ecosystem, when intestinal microbiota imbalance can result in a decline of the pivotal functions of the gut, subsequently elevating the risk of UC onset.

The human digestive system hosts a diverse and intricate microbial community that profoundly impacts disease progression, particularly in maintaining intestinal homeostasis, modulating immune responses, and regulating inflammation (Wang et al., 2020). The healthy adult gut harbors over 100 trillion bacteria, encompassing more than 1,000 species with varying levels of abundance (Lozupone et al., 2012). However, disruptions in the gut microbiota can precipitate various health disorders, including inflammatory and immune-related conditions (Ding et al., 2024). Evidence indicates that fecal transplantation effectively restores microbial equilibrium in the intestine (Zhao et al., 2021). Although the exact mechanisms driving these responses remain unclear, accumulating data implicates intestinal microbiota dysregulation as a significant contributor to UC pathogenesis (Zhu et al., 2022). The intestinal flora was significantly less rich and diverse in UC patients than in healthy control subjects. Previous studies have shown that, compared with healthy people, the intestinal environment of UC patients is characterized by a reduced presence of *Firmicutes* and an increased prevalence of *Bacteroidetes* organisms, as well as *facultative anaerobes* (Hansen et al., 2010). Notably, the abundance of *Enterobacteriaceae*, particularly *Escherichia coli* and *Shigella*, correlates with heightened intestinal inflammation. In contrast, there is a concurrent decrease in the representation of *Lachnospiraceae* and *Ruminococcaceae*, which are typically more abundant in healthy gut microbiomes (He et al., 2021). Conversely, promoting the growth of *Lactobacillus* and *Akkermansia* while suppressing *Vibrio erysipelas* may alleviate UC symptoms (Ke et al., 2021). The disruption of intestinal microbiota is strongly associated with intestinal inflammation. The immune system of the intestinal mucosa employs multiple mechanisms to protect the host from pathogenic infections and minimize tissue damage arising from innate and adaptive immune responses (Jackson and Theiss, 2020). Insufficient regulation of these immune processes can lead to chronic inflammation (Koboziev et al., 2014). Concurrently, evidence suggests that microbial dysbiosis and pathogenic bacterial overgrowth compromise the intestinal mucosa, weakening its structural integrity (Feng et al., 2020). Research highlights that chitosan ameliorates UC in mice by enhancing intestinal barrier function, increasing beneficial *Cyanobacteria* and *Lactobacilli* populations, and restoring microbial balance in the gut (Wang et al., 2019). Similarly, 16S rRNA sequencing demonstrated that eugenolide mitigated UC by repairing epithelial integrity and modulating gut microbiota and metabolites. Furthermore, fecal transplantation can alleviate colitis by elevating acetate levels and adjusting the thick-walled bacteria-to-*Bacteroidetes* ratio (Wang et al., 2020). Kaempferol can modify the gut microbiome by increasing the *Firmicutes*-to-*Bacillus mimicus* ratio while reducing *Aspergillus* abundance in UC mice (Qu et al., 2021). Collectively, these findings highlight the potential of intestinal microbiota regulation as a therapeutic strategy for UC.

At present, there are many strategies for the treatment of UC, encompassing drug therapy, fecal bacteria transplantation, surgical treatment and diet management, which are mainly drug therapy, such as immunosuppressants, corticosteroids and tumor necrosis factor antagonists (Schreiber et al., 2023). While these medications provide symptomatic relief, many patients eventually experience diminished efficacy or adverse effects, imposing significant physical and financial burdens (Kobayashi et al., 2020). This highlights the necessity of expanding therapeutic options for UC. Notably, traditional Chinese medicine has gained clinical recognition for managing chronic conditions like UC due to its significant therapeutic efficacy and minimal side effects (Zhang et al., 2013). Saussurea costus (SC) is a traditional Chinese herbal medicine has been documented to substantially promote the healing of duodenal ulcer and inhibit peptic ulcer in animals. Its bioactive constituents, including alantolactone, dehydrocostunolide lactone, and costunolide, exhibit pronounced anti-inflammatory properties, effectively ameliorating UC symptoms (Cai et al., 2022). Moreover, evidence suggests that costunolide modulates gut microbiota by increasing *Firmicutes* and *Actinobacteria* while decreasing *Bacteroidetes* and *Proteobacteria* (Mao et al., 2022), underscoring SC potential as a therapeutic candidate for UC.

In this study, the 16S rRNA gene sequencing technique was employed to analyze alterations in the intestinal microflora of UC mice following treatment and to assess the capacity of SC to mitigate inflammation and restore the integrity of the damaged intestinal mucosa.

## 2 Materials and methods

### 2.1 Animals

C57BL/6J mice (6–8 weeks old) were procured from SBF Biotechnology Co., Ltd. [License No. SCXK(Beijing) 2019-0010] and housed under specific pathogen free (SPF) conditions at Yunnan University of Traditional Chinese Medicine (R-062021115). All housing and experimental protocols adhered to the ethical guidelines established by the Animal Care and Usage Committee at Yunnan University of Traditional Chinese Medicine (Kunming, China, permission No. SYXK 2017-0005).

### 2.2 Modeling and grouping

In a controlled environment (20 ± 2°C, 40–60% humidity, 12 hour light/dark cycle), mice were housed under pathogen-free conditions with access to a standard diet and water. After one week of acclimatization, the C57BL/6J mice were randomly allocated into five groups using a random number table: control, model, and three SC treatment groups. Colitis was induced in all groups except the control by administering 3% DSS (160110, MP, USA) for seven days. SC was subsequently administered via gavage at doses of 0.4 g/kg/day, 0.8 g/kg/day, and 1.6 g/kg/day from days 8 to 14.

### 2.3 Blood biochemical assay

Serum analysis was performed using alanine aminotransferase (ALT) (C009-2-1), aspartate aminotransferase (AST) (C010-2-1), blood urea nitrogen (BUN) (C013-2-1), creatinine (C011-2-1), uric acid (UA) (C012-2-1), and lactate dehydrogenase (LDH) (A020-2-2) assay kits, all sourced from Nanjing Jiancheng, China.

### 2.4 Ultra-high performance liquid chromatography-quadrupole time-of-flight mass spectrometry analysis

The preparation of SC decoction involved combining SC with water at an 8:1 ratio by volume, followed by boiling three times for 30 minutes each. The resulting filtrates were collected separately in three batches. The specimens were then sent to Shanghai Biotree Biomedical Biotechnology Co., Ltd. for analysis via UHPLC-QTOF-MS.

### 2.5 Disease activity index

The disease activity score, used to evaluate UC severity in mice, was derived from measurements of body weight changes, fecal consistency, and occult blood levels. The scoring system included weight loss percentages (0: none; 1: 1%-5%; 2: 5%-10%; 3: 10%-15%; 4: >15%), fecal consistency (0: normal; 2: semi-liquid; 4: liquid), and blood detection (0: negative; 2: positive occult blood; 4: visible blood).

### 2.6 Hematoxylin-eosin(HE) staining

Colons were preserved in 4% paraformaldehyde for 24 hours before paraffin embedding. Subsequently, 5-μm-thick colonic tissue sections were prepared and stained with HE (G1120, Solarbio, China) for histological examination. To evaluate the degree of inflammation present in the samples, a histological colitis scoring system was utilized. This scoring system assigns values ranging from 0 to 12, representing the total score derived from individual cumulative assessments of severity, extent, damage, inflammation, and regeneration, each of which is rated on a scale of 0 to 4.

### 2.7 Real-time PCR assay

Total RNA was isolated from colonic tissues using RNAiso Plus (9109, Takara, China) following the manufacturer’s instructions. cDNA synthesis was carried out with a reverse transcription kit (11141ES60, Yeasen, China). RT-PCR was subsequently performed using SYBR^®^ Premix-Ex TagTM (11202ES08, Yeasen, China) combined with specific primers listed in **Supplementary Table 1**. Amplification and detection were conducted on an ABI QuantStudio 5 Real-Time PCR System (Applied Biosystems, Darmstadt, Germany). Relative mRNA expression levels of target genes were normalized to β-actin, and quantification was calculated using the 2−^ΔΔCT^ method.

### 2.8 Immunofluorescence assay

Following the removal of wax from the paraffin sections, colonic tissue samples were treated overnight at 4°C with primary antibodies, specifically anti-occludin and ZO-1, diluted at 1:1000. After incubation, the sections were rinsed with PBST (phosphate-buffered saline with Tween 20) to prepare for subsequent steps. A fluorescently labeled secondary antibody (A21207, A11034, Invitrogen, USA, 1:1000) was applied for 1 hour. The samples were then washed with PBS, stained with DAPI, and imaged using microscopy.

### 2.9 Western blotting assay

Colon tissues were lysed with RIPA lysis buffer (P0013B, Beyotime, China) supplemented with protease and phosphatase inhibitors. Protein concentrations were quantified using a BCA protein assay kit (P0010, Beyotime, China). Proteins, standardized to equal concentrations, were resolved on 8% or 10% SDS-PAGE gels and transferred onto PVDF membranes (IPVH00010, Millipore, USA). Membranes were blocked for one hour in 5% milk-TBST solution and incubated overnight at 4°C with primary antibodies, including β-Actin (GB11001, Rabbit monoclonal, 1:5000, Servicebio), ZO-1 (21773-1, Rabbit polyclonal, 1:5000, Proteintech), and Occludin (27260-1, Rabbit polyclonal, 1:8000, Proteintech). Following incubation with HRP-conjugated secondary antibodies, immunoreactivity was visualized using an ECL detection kit (220616-60, Western Bright TM ECL, Advansta). Images were acquired using the i-Bright™ CL1500 Imaging System (Thermo Fisher Scientific).

### 2.10 16S rRNA gene sequencing

Fresh fecal samples were collected for genomic DNA extraction, followed by PCR amplification, product purification, and standardized quantification to ensure consistent amplification efficiency and precision. Library construction was performed, with quality control measures implemented to validate library integrity. Sequencing was conducted using the Illumina 6000 system, employing high-throughput methods to generate raw data. Subsequent base calling converted raw data into original sequences, which were further analyzed for relevant information.

### 2.11 Statistical analysis

Results were derived from three independent, replicated experiments. Data are presented as mean ± standard error of the mean and analyzed using GraphPad Prism 9 Statistical significance was assessed via one-way analysis of variance (ANOVA), revealing differences with *P* < 0.05.

## 3 Results

### 3.1 Screening active components of SC extract with UHPLC-QTOF-MS

The primary constituents of SC were analyzed using UHPLC-QTOF-MS in both positive and negative modes, a total of 291 components were obtained. Among them, the top 20 substances in each mode ranked by peak area (**Table 1**) were emphasized as potential candidates for alleviating UC.

**Table 1:** Identification of components of SC extract by UHPLC-QTOF-MS analysis.

MS2Components	MS2 ppm		Electric	mzmed	rtmed	Peak area
Proline		3.308	+H	116.070	32.392	567,824,324.952
Phenylalanine		0.570	+H	166.086	36.758	320,576,032.921
6-Hydroxy-5a-methyl-3,9-bis(methylene)decahydronaphtho[1,2-b]furan-2(3H)-one		0.577	+H	231.138	494.794	264,683,597.452
Isoleucine		1.153	+H	132.102	35.771	229,636,631.912
Azuleno[5,6-c]furan-1(3H)-one, 4,4a,5,6,7,7a,8,9-octahydro-4,8-dihydroxy-6,6,8-trimethyl-		0.329	+H	249.148	218.325	216,079,511.433
Lucidenic acid M		0.549	+H	463.303	656.054	166,089,835.562
Pyroglutamic acid (not validated, isomer of 88)		2.482	+H	130.050	32.884	161,684,203.871
2-(8-hydroxy-4a,8-dimethyl-1,2,3,4,5,6,7,8a-octahydronaphthalen-2-yl)prop-2-enoic acid		1.562	+H	235.169	590.907	113,827,871.523
(+)-Alantolactone		1.870	+H	233.153	360.994	90,370,539.383
Isospathulenol		0.756	+H	221.190	393.189	75,933,548.281
Grandisin		1.003	+H	415.211	463.705	67,038,940.292
Tyramine		3.843	+H	121.064	652.852	63,505,451.671
7-[(6-hydroxy-2,5,5,8a-tetramethyl-1,4,4a,6,7,8-hexahydronaphthalen-1-yl)methoxy]chromen-2-one		1.047	+H	365.207	124.125	60,367,360.403
Tricyclohumuladiol		0.586	+H	239.200	393.189	57,192,464.601
Piperdial		1.304	+H	251.164	289.339	53,065,976.975
1alpha-Hydroxyarbusculin A		1.614	+H	267.159	101.998	52,023,771.347
Saussurea lactone		0.565	+H	235.169	196.806	43,462,366.613
5-[6-(3-hydroxy-4-methoxyphenyl)-1,3,3a,4,6,6a-hexahydrofuro[3,4-c]furan-3-yl]-2-methoxyphenol		0.928	+H	341.138	46.914	35,964,398.593
Acorenone		1.024	+H	221.190	296.627	31,447,229.592
Santonin		3.016	+H	247.132	305.813	34,712,692.504
Citric acid		2.285	-H	191.020	32.497	225,039,694.920
Dicaffeoyl quinic acid		0.798	-H	515.120	52.937	112,069,729.628
5-OXO-D-PROLINE		4.485	-H	128.035	33.590	79,350,870.341
L-PROLINE		2.886	-H	114.056	239.569	68,610,948.208
S(8-8)S hexoside		2.127	-H	579.208	148.082	49,722,754.338
Azuleno(5,6-c)furan-1(3H)-one, 4,4a,5,6,7,7a,8,9-octahydro-3,4,8-trihydroxy-6,6,8-trimethyl-		2.043	-H	281.139	129.069	42,057,027.044
13-HODE		1.826	-H	295.227	562.933	41,597,968.296
2-METHYLMALEATE		4.193	-H	129.019	36.418	38,934,031.972
MALEIC ACID		2.025	-H	115.004	43.099	38,484,702.759
Azelaic acid		1.029	-H	187.098	156.282	34,572,300.184
Caffeic acid		0.190	-H	179.035	56.526	31,822,221.460
Salicylic acid		1.290	-H	137.025	160.467	18,906,505.040
Abscisic acid		0.433	-H	263.129	185.472	18,729,527.188
D-Gluconic acid		0.268	-H	195.051	32.497	17,764,555.686
4-[4-[hydroxy-(4-hydroxy-3-methoxyphenyl)methyl]-3-(hydroxymethyl)oxolan-2-yl]-2-methoxyphenol		1.829	-H	375.144	109.445	17,024,334.113
Curcumenol		0.489	-H	233.155	591.595	16,528,296.196
p-Hydroxybenzaldehyde		1.429	-H	121.030	78.930	14,773,033.972
Aconitic Acid		2.851	-H	173.009	32.497	14,627,700.830
Protocatechuic acid		3.180	-H	153.020	42.135	13,898,210.686
Isokobusone		1.378	-H	221.155	404.209	13,476,977.966

### 3.2 Treatment with SC alleviates UC

**Figure 1:**
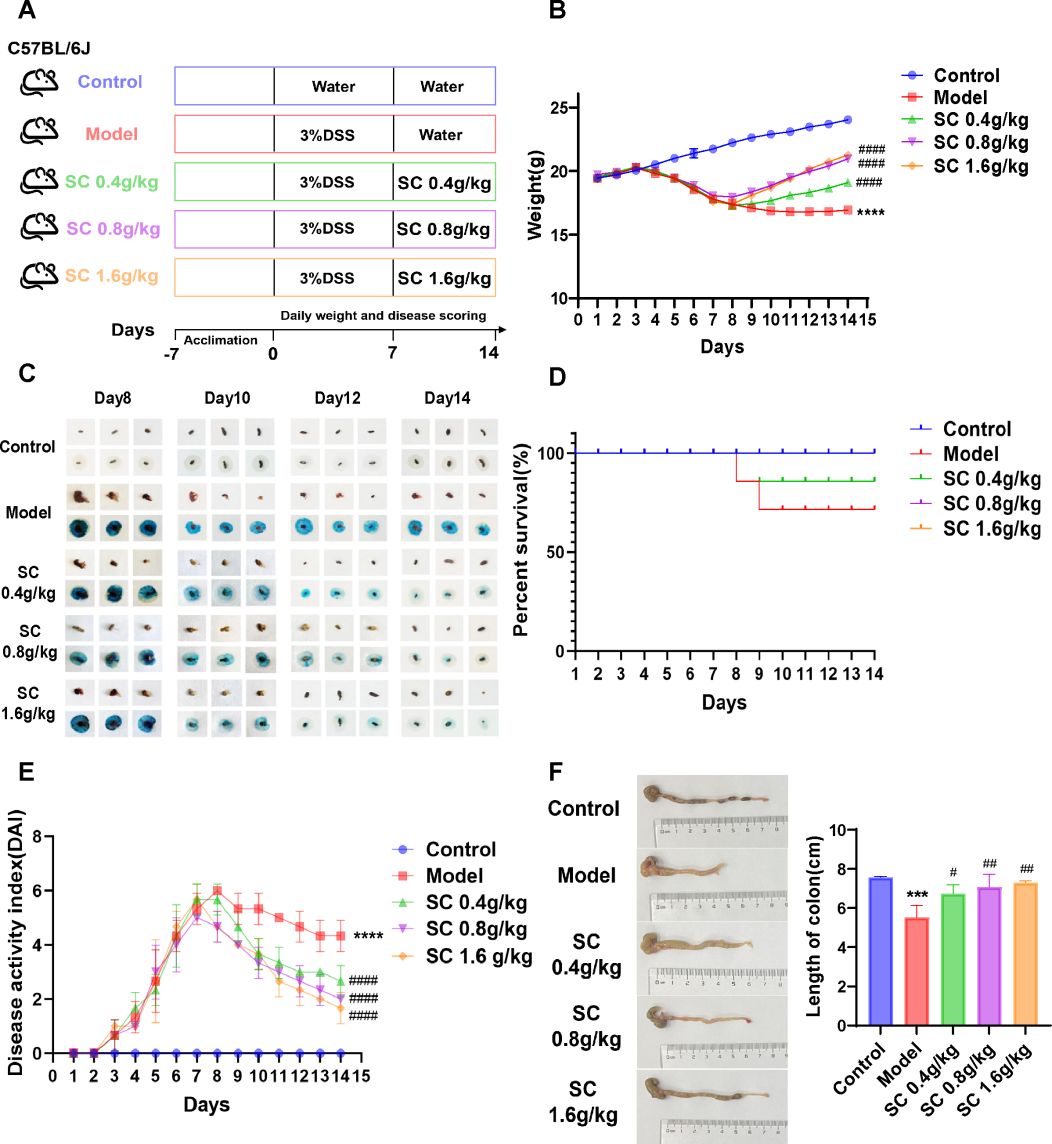
SC significantly improved the disease characteristics of UC mice. **(A)** The design of experiments in mice; **(B)** weight changes; **(C)** Fecal occult blood; **(D)** Survival rate of mice; **(E)** DAI scores; **(F)** Colonic length. (n=3 per group, ***P < 0.001, ****P < 0.0001 vs. control; ^#^ *P* < 0.05, ^##^ *P* < 0.01, ^####^ *P* < 0.0001 vs. model).

To assess the therapeutic efficacy of SC in colitis, DSS-induced mouse model of colitis was developed, and SC was administered orally at different doses (**Figure 1A**). Body weight and fecal occult blood were monitored throughout the experiment, revealing that SC treatment significantly mitigated body weight loss (**Figure 1B**) and reduced fecal occult blood levels (**Figure 1C**) in colitis mice. Notably, the survival rate of mice receiving a high dose of SC was significantly lower than that of the other groups (**Figure 1D**). The DAI score, calculated based on fecal occult blood, stool consistency, and weight loss, demonstrated substantial improvement across all SC-treated groups, with the high-dose group showing the greatest therapeutic benefit (**Figure 1E**). Additionally, SC administration at different doses resulted in a marked increase in colon length compared to the model group (**Figure 1F**). Collectively, the findings indicate that SC effectively alleviates UC symptoms.

### 3.3 SC improves histological morphology of the UC

**Figure 2:**
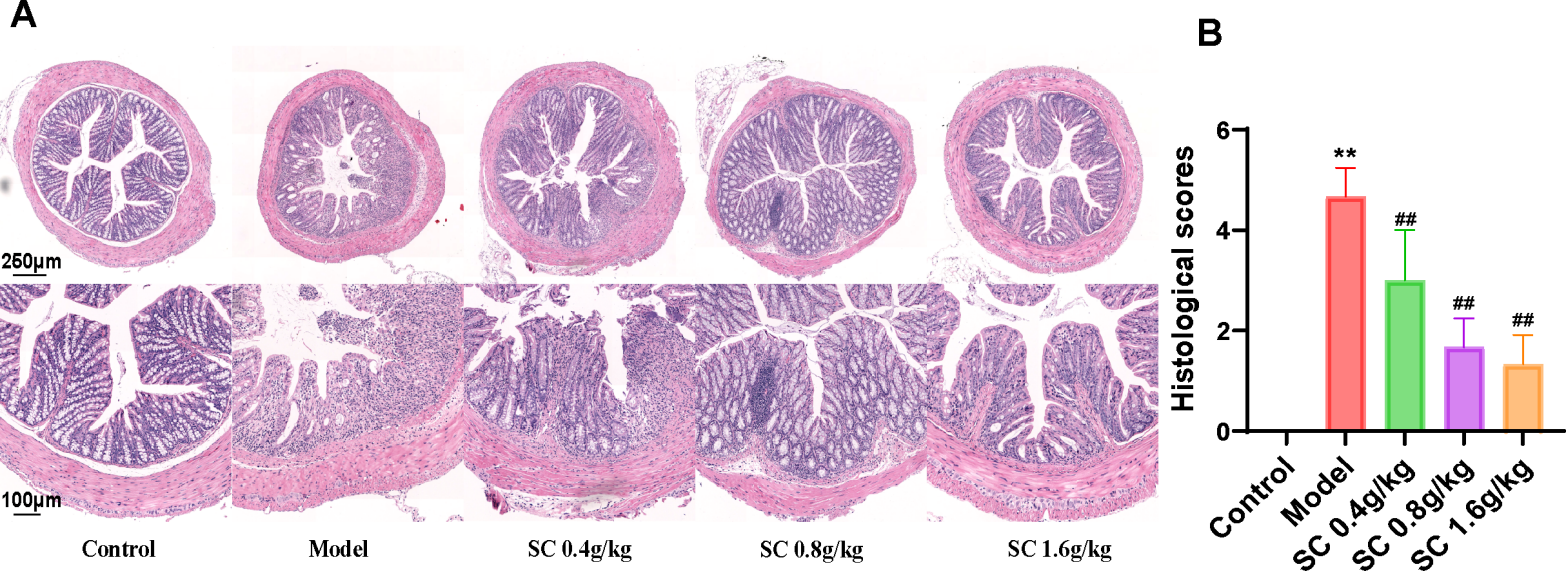
SC restored pathological damage of colon tissue. **(A)** HE-stained sections of the distal colon of differently treated mice; **(B)** histological scores. (n=3 per group, **P < 0.01 vs. control; ^##^ *P* < 0.01 vs. model).

The protective effects of SC on intestinal barrier integrity were assessed by analyzing histological damage in the mouse colon using HE staining. DSS-induced mice exhibited substantial inflammation and tissue injury, characterized by irregular and disrupted colonic crypt structures, loss of goblet cells, infiltration of inflammatory cells, and elevated pathological scores. Treatment with SC at different doses markedly alleviated these abnormalities, with medium and high doses demonstrating the most significant improvements (**Figures 2A, B**).

Based on the above findings, suggesting that SC has a potential therapeutic effect on UC, we then explored the toxicity characteristics of high-dose SC, serum biochemical tests indicated no apparent liver and kidney toxicity in the SC-treated mice (**Supplementary Figures S1A–F**). These findings indicate that SC appears to be safe in colitis mice.

### 3.4 SC repairs the intestinal barrier and weakens the inflammatory damage of UC

**Figure 3:**
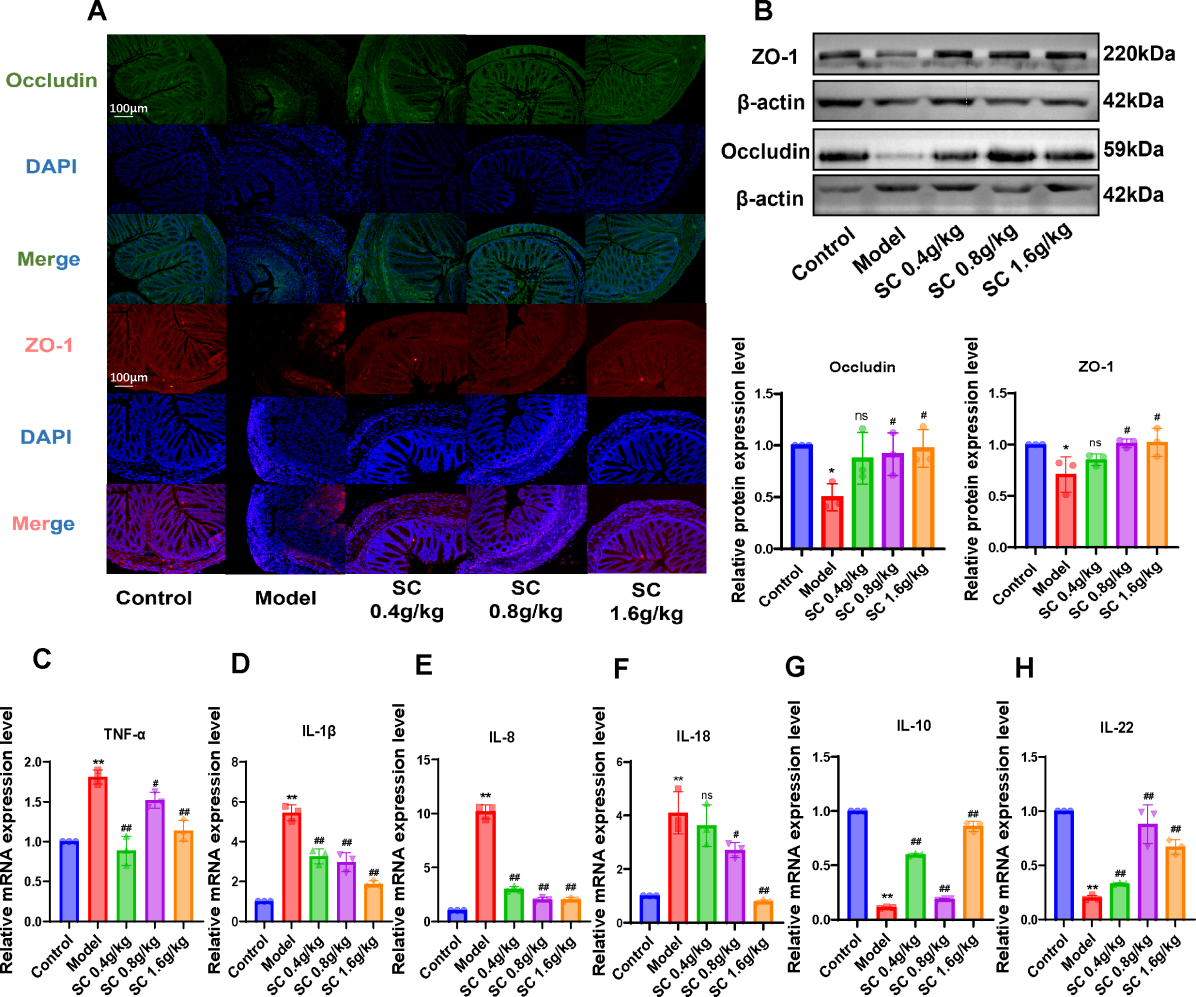
The administration of SC can enhance the restoration of the intestinal barrier and suppress the inflammatory response. **(A)** The expression of intestinal barrier related ZO-1 and Occludin were detected by immunofluorescence. **(B)** The expression of intestinal barrier related ZO-1 and Occludin were detected by western blot. **(C–H)** The relative mRNA expression levels of TNF-α, IL-1β, IL-8, IL-18, IL-10, and IL-22 in colon tissue of mice were detected by RT-PCR. (n=3 per group, ns, nonsignificant *P < 0.05**P < 0.01 vs. control; ^#^ *P* < 0.05^##^ *P* < 0.01 vs. model).

To investigate the effects of SC on intestinal barrier dysfunction and inflammation in colitis mice, tight junction proteins ZO-1 and Occludin in colonic tissues were analyzed using immunofluorescence. The model group exhibited a marked reduction in ZO-1 and Occludin expression, while SC administration at different doses significantly restored their levels in colonic tissues (**Figure 3A**). Consistent results were confirmed through western blot analysis, demonstrating that SC reversed the downregulation of ZO-1 and Occludin proteins expression in colitis mice (**Figure 3B**).

Additionally, inflammatory markers in colonic tissues were evaluated, revealing increased mRNA expression of TNF-α, IL-1β, IL-8 and IL-18, alongside decreased IL-10 and IL-22 levels in the model group. SC treatment significantly alleviated inflammatory responses in colonic tissues (**Figures 3C-H**). These results indicate that SC may ameliorate UC by restoring intestinal barrier integrity and suppressing inflammation.

### 3.5 SC modulates the gut microbiota in UC

**Figure 4:**
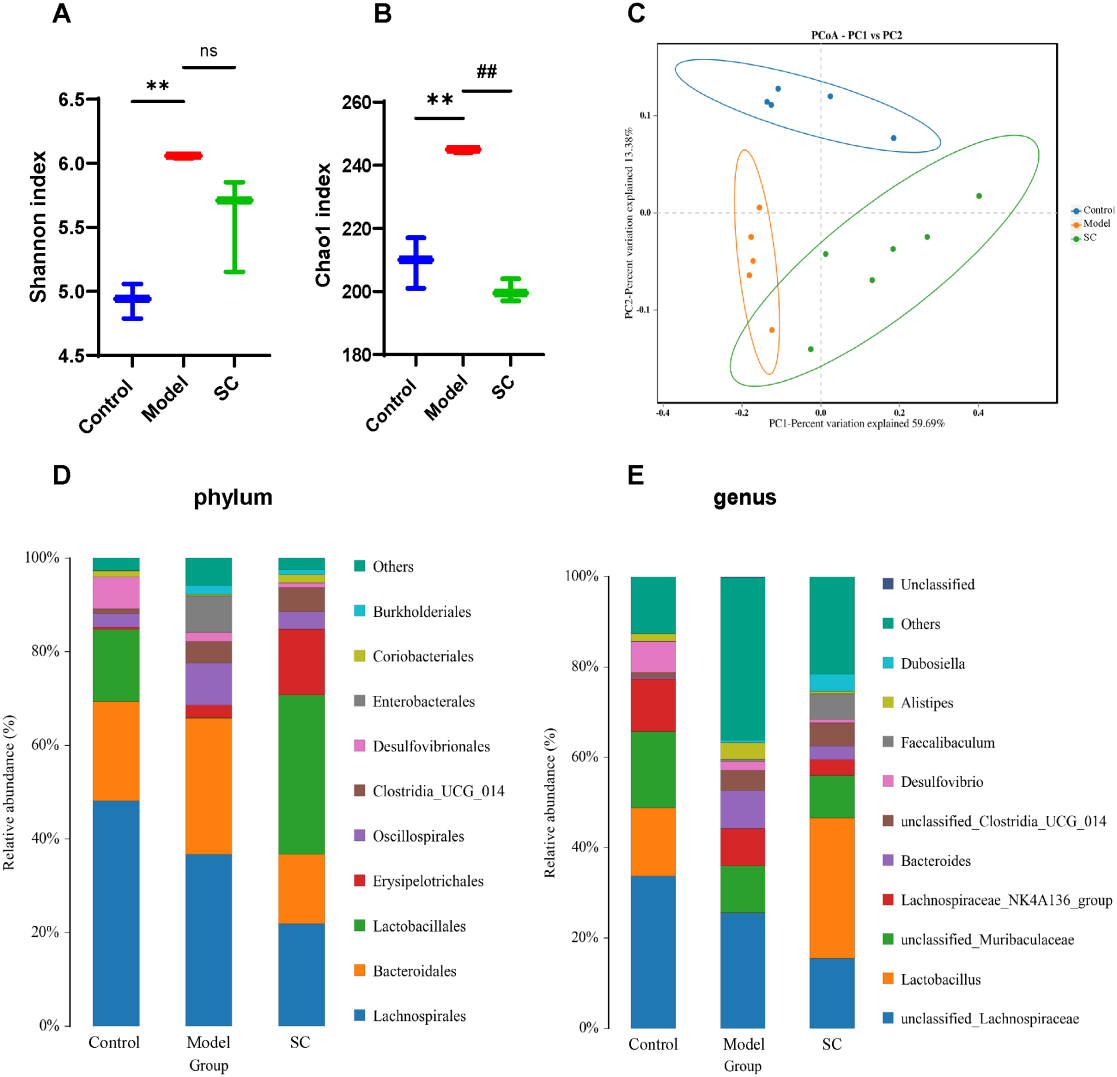
SC modulates gut microbiota in UC. **(A)** Shannon index; **(B)** Chao 1 index; **(C)** Principal component analysis **(D)** The proportions of the phylum; **(E)** The proportions of the genus; (n=5 per group, ns, nonsignificant **P < 0.01 vs. control; ^##^ *P* < 0.01 vs. model.

To further assess whether SC regulates intestinal microbiota in UC, we used 16s rRNA gene sequencing to analyze intestinal microbiota in colorectum contents. The Shannon and Chao1 indices were employed to characterize the alpha-diversity, thereby assessing species richness and evenness. The Shannon and Chao1 indices were significantly elevated in the DSS-induced model group, whereas SC-treated mice showed a decreasing trend (**Figures 4A, B**). Principal coordinate analysis (PCoA) is employed to measures beta-diversity and evaluate the differences or similarities among microbiota communities. The PCoA score plot demonstrated that the three groups exhibited significant distinctions in their microbiota community structures (**Figure 4C**).

Next, we observed the bacteria in phylum and genus levels among the control group, model group, and SC group. At the phylum level, the predominant bacterial phylum in the gut microbiota were *Firmicutes* and *Bacteroidetes*. The findings indicated a rising in *Proteobacteria*, alongside a decline in *Firmicutes* observed in mice with UC. After the intervention with SC, a decrease in the levels of *Proteobacteria* was noted, paired with an increase in *Firmicutes* (**Figure 4D**), at the genera level, the colorectal microbiota was dominated by *unclassified_Lachnospiracease*, *unclassified_Muribaculaceae*, *Lactobacillus*, *Lachnospiraceae_NK4A136*, *Baceroides*. In contrast to the control group, the DSS group displayed an increased proportion of *Bacteroides*, *Alistipes*, and several other species within their microbiota. In contrast, a reduction in the relative abundance of *Lactobacillus* was observed. Following treatment with SC, there was an increase in the relative abundance of *Lactobacillus*, *unclassified_Clostridia_UGG_014*, *Faecalibaculum*, and *Dubosiella*, while *Bacteroides* and *Alistipes* showed a decreased relative abundance (**Figure 4E**).

### 3.6 KEGG/COG annotation analysis

**Figure 5:**
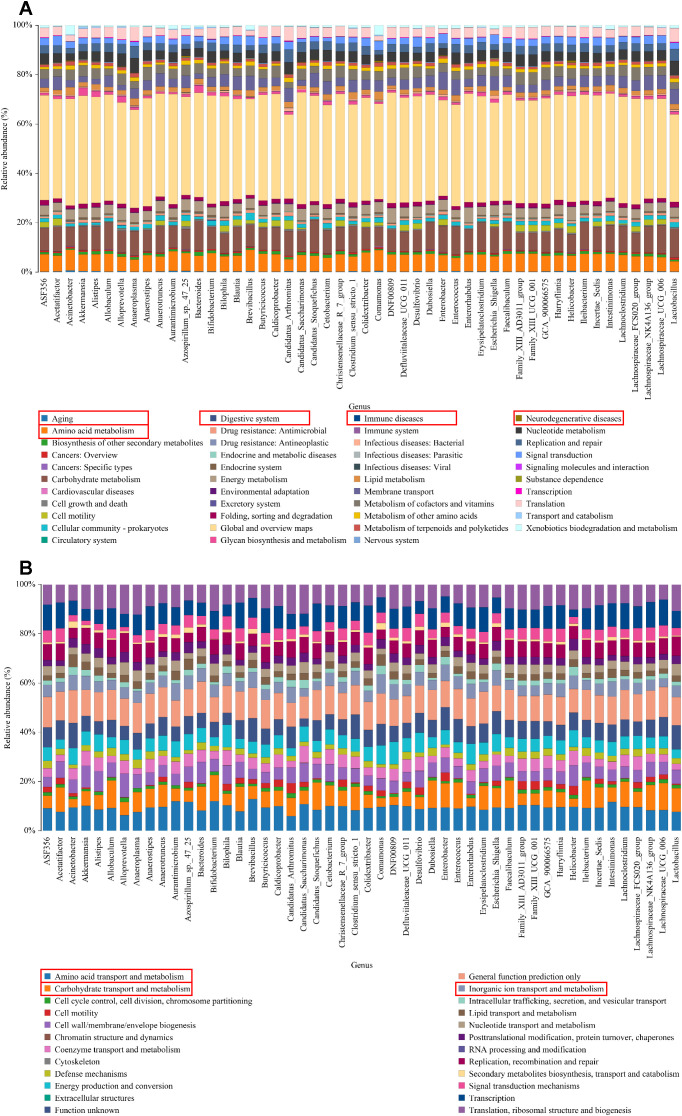
KEGG/COG analysis. **(A)** The KEGG enrichment analysis of the genus; **(B)** The COG function prediction analysis of the genus.

KEGG pathway analysis revealed significant variations in functional genes within microbial communities associated with metabolic pathways. Concurrently, COG functional prediction identified the distribution and abundance of sequences within the sample. KEGG metabolic pathway analysis showed that the regulation of intestinal microbiota in UC mice may be closely related to aging, digestive system diseases, immune diseases, neurodegenerative disease and amino acid metabolism (**Figure 5A**). COG function prediction analysis suggested that the intestinal microbiota function of UC mice was closely related to amino acid transport metabolism, carbohydrate transport and metabolism and inorganic ion transport and metabolism (**Figure 5B**). These results propose novel perspectives for investigating the role of SC in alleviating UC, warranting further study.

## 4 Discussion

Inflammation is a persistent characteristic observed in UC, which is classified as a chronic inflammatory bowel condition. Substantial evidence indicates elevated pro-inflammatory cytokines in both UC patients and DSS-induced UC mice (Shao et al., 2024). Traditional Chinese medicine has garnered attention for its anti-inflammatory properties, with Saussurea, first documented in *Shennong’s Herbal Classics* as a treatment for diarrhea and dysentery, emerging as a focus of interest. SC, native to Yunnan Province and recognized as a geo-authentic crude drug, contains bioactive compounds such as terpenoids, alkaloids, and flavonoids, which exhibit potent pharmacological effects, including anti-inflammatory, anti-tumor, and anti-ulcer activities (Elnour and Abdurahman, 2024). Some studies indicate that the costunolide, the main active component of the terpenoids of saussurea, targets NLRP3 to inhibit inflammasome activation in inflammatory diseases (Xu et al., 2023). Additionally, costiolactone can mitigate DSS-induced colitis by reducing pro-inflammatory cytokines TNF-α, IL-6, IL-1β and IFN-γ, which may be related to the NF-κB, STAT1/3 and Akt signaling pathways (Xie et al., 2020) and costunolide also can restore Th17/Treg balance in colons, mesenteric lymph nodes, and spleen, further diminishing pro-inflammatory cytokine levels in colitis mice (Lv et al., 2021). Among Saussurea’s active components, sesquiterpene lactone has also demonstrated anti-inflammatory effects in alleviating colitis. This mechanism may involve co-regulation of the MAPK and Nrf2/Hmox-1 signaling pathways[22b]. In our study, SC significantly reduced TNF-α, IL-1β, IL-8, and IL-18 levels in UC mice, reinforcing its anti-inflammatory potential against DSS-induced colitis.

Repairing intestinal barrier damage is recognized as a fundamental strategy in managing UC (Mansouri et al., 2025). Tight junctions, essential structures connecting intestinal epithelial cells, maintain intercellular sealing under physiological conditions, preventing the translocation of pathogenic microorganisms, antigens, and luminal substances into the lamina propria of the intestinal mucosa. However, Disruption of these tight junctions allows harmful entities to penetrate the lamina propria, thereby triggering severe inflammatory responses and aggravating colitis progression (Matar et al., 2024). Therefore, regulating the gut microbiota may be the key to alleviating UC. The imbalance of gut microbes may be proportional to the progression of UC. Studies on DSS-induced colitis in mice have demonstrated a marked reduction in beneficial bacteria, such as *Lactobacillaceae* and *Lachnospiraceae*, accompanied by intense inflammatory responses (Li et al., 2024). A human cohort data indicated a decrease in bacterial fucosylation levels among IBD patients, correlating with intestinal inflammation, and reveal that mice with deficient *Bacteroides* exhibiting lower surface fucosylation are more susceptible to colitis (Lei et al., 2024). Furthermore, *Lactobacillus* has been shown to elevate IL-10 levels by modulating the development and maturation of regulatory T cells in colitis (Wu et al., 2022). Collectively, the evidence highlights a significant relationship between gut microbiota and colitis pathogenesis.

In addition, intestinal microbes are integral to intestinal barrier repair processes. Studies revealed that walnut peptides restored microbial balance by reducing harmful bacteria, such as *Helicobacter pylori* and *Bacteroides*, while promoting beneficial bacteria, including *Candidatus_Saccharimonas*. Mechanistic insights have highlighted their anti-inflammatory properties and barrier-repair capabilities, showing that *Bacteroides* and *Oscillospiraceae_UCG-005* negatively correlate with pro-inflammatory factors (IL-1β, IL-6 and TNF-α) and positively correlate with anti-inflammatory factors and barrier-associated proteins (ZO-1, and Occludin) (Hong et al., 2023). Furthermore, *Lactobacillus*, a dominant beneficial genus, has demonstrated efficacy in restoring intestinal barrier function, potentially by suppressing *Bacteroides* and *Erysipelatoclostridium*, thereby normalizing gut microbiota composition (Zhao et al., 2024). Recently, the regulation of intestinal microbiota through traditional Chinese medicine has attracted increasing interest. Rosavin selectively modulated gut microbiota by reversing DSS-induced microbial dysbiosis and increasing beneficial microbes such as *Lactobacillus* and *Akkermansia* (Wang et al., 2024). Huangqin decoction enhanced the prevalence of *Lactobacillus* and *Firmicutes*, while significantly reducing *Treponema* and *Bacteroides* populations (Zheng et al., 2022). The Xianglian pills, a traditional formulation combining Saussurea and Coptis, regulated microbial succinate production and repaired intestinal barrier damage, effectively mitigating UC symptoms (Liu et al., 2023). A comparable effect was observed with the Xianglian Zhixie Tablet, another classical prescription incorporating Saussurea, which promoted MUC-2, Occludin, and ZO-1 expression to restore intestinal barrier integrity. This mechanism involved boosting beneficial bacterial populations while reducing harmful microbial adherence in the gut (Li et al., 2023). Collectively, these studies highlight the therapeutic potential of Saussurea in modulating gut microbiota and promoting intestinal barrier repair.

Focusing on the role of intestinal microbiota, the study investigated the mechanism by which SC alleviated UC and its association with microbiota modulation in UC mice. SC treatment significantly reduced *Proteobacteria* and *Bacteroides* populations while promoting the growth of lactic acid bacteria, consistent with previous research and providing robust evidence for this therapeutic strategy. Notably, the ability of SC to regulate gut microbiota appeared closely linked to gastrointestinal, endocrine, and immune system disorders, as well as aging, amino acid metabolism, and cardiovascular conditions. Furthermore, SC impact may extend to pathways involving amino acid transport, synthesis of cellular structures such as walls and membranes, coenzyme transport, and signal transduction mechanisms.

## 5 Conclusions

In conclusion, our study demonstrates that SC effectively mitigates DSS-induced UC symptoms, suppresses intestinal inflammation, strengthens intestinal barrier function, and modulates gut microbiota composition. The findings highlight the potential of SC as a therapeutic strategy for managing UC.

## Data Availability

The datasets presented in this study can be found in online repositories. The names of the repository/repositories and accession number(s) can be found below: https://www.ncbi.nlm.nih.gov/, PRJNA1185471.
