# Supplementary material for: Saussurea costus alleviates ulcerative colitis by regulating the gut microbiota and improving intestinal barrier integrity
Source: Front Cell Infect Microbiol. 2025 Jan 28;15:1528578. doi: 10.3389/fcimb.2025.1528578 (PMC11810970; doi:10.3389/fcimb.2025.1528578)
Supplement: Supplementary file 1 [file DataSheet1.docx]

Supplementary Material

# Supplementary Figure S1


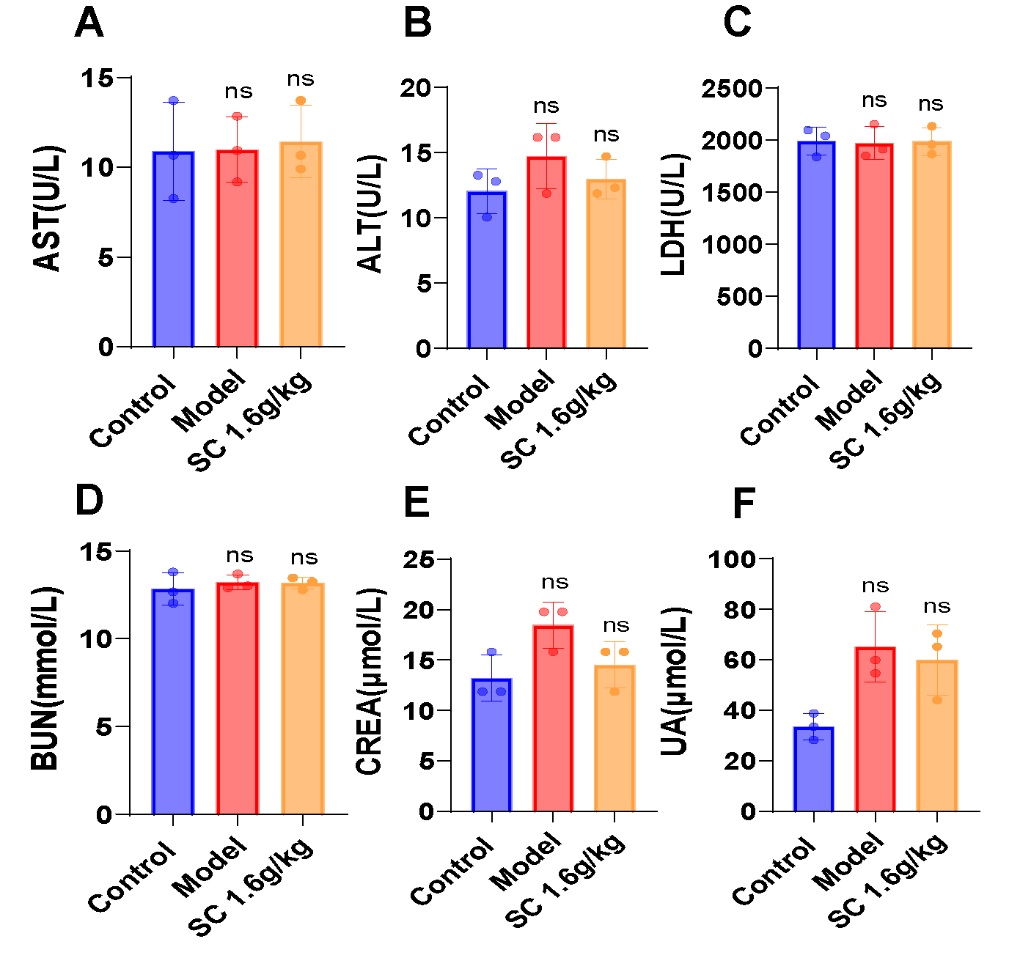


**Evaluation of liver and kidney toxicity** (A) Aspartate aminotransferase (AST); (B), Alanine aminotransferase (ALT); (C) Lactate dehydrogenase (LDH); (D) Blood urea nitrogen (BUN); (E) Creatinine (CREA); (F) Uric acid (UA) (n=3 per group, ns nonsignificant)
