# Supplementary material for: Saussurea costus alleviates ulcerative colitis by regulating the gut microbiota and improving intestinal barrier integrity
Source: Front Cell Infect Microbiol. 2025 Jan 28;15:1528578. doi: 10.3389/fcimb.2025.1528578 (PMC11810970; doi:10.3389/fcimb.2025.1528578)
Supplement: Supplementary file 2 [file Table1.docx]

Supplementary Material

# Supplementary Table 1

Table 1 Sequences of q-PCR primers

| Names | Sequences |
| --- | --- |
| TNF-α | Forward primer:5'→GACGTGGAACTGGCAGAAGAG→3'  Reverse primer:5'→TTGGTGGTTTGTGAGTGTGAG→3' |
| IL-1β | Forward primer:5'→TGCCACCTTTTGACAGTGATG→3'  Reverse primer:5'→TGATGTGCTGCTGCGAGATT→3' |
| IL-8 | Forward primer:5'→GGCCCAATTACTAACAGGTTCC→3'  Reverse primer:5'→TCTCTTGTTCTCAGGTCTCCCA→3' |
| IL-10 | Forward primer:5'→GCTGTCATCGATTTCTCCCCT→3'  Reverse primer:5'→GACACCTTGGTCTTGGAGCTTAT→3' |
| IL-18 | Forward primer:5'→TCACTTCTCCCCTGTGGTGT→3'  Reverse primer:5'→GGTCCAGCTGTGCCAGTC→3' |
| IL-22 | Forward primer:5'→GCTCAGCTCCTGTCACATCA→3'  Reverse primer:5'→CAGTTCCCCAATCGCCTTGA→3' |
| β-Actin | Forward primer:5'→CATTGCTGACAGGATGCAGAAGG→3'  Reverse primer:5'→TGCTGGAAGGTGGACAGTGAGG→3' |
